# The Novel Role of Cytomorphology from Ultrasound-Guided Fine Needle Aspiration Cytology in Evaluating the Status of Prognostic Factors including Estrogen Receptor, Progesterone Receptor and HER2 in Breast Cancer

**DOI:** 10.1155/2022/6302751

**Published:** 2022-03-14

**Authors:** Yunzhu Li, Shijun Jia, Yuqi Yao, Yehan Zhou, Shurong Li, Jiayu Li, Yang Liu

**Affiliations:** ^1^Department of Pathology, Sichuan Cancer Hospital & Institute, Sichuan Cancer Center, School of Medicine, University of Electronic Science and Technology of China, Chengdu, China; ^2^Development and Regeneration Key Lab of Sichuan Province of Chengdu Medical College, Chengdu, 610500 Sichuan, China

## Abstract

It is well established that estrogen receptor (ER), progesterone receptor (PR), and human epidermal growth factor receptor (HER2) could be regarded as prognostic factors in breast cancer. Ultrasound-guided fine needle aspiration cytology (FNAC) has revolutionized the management of cancers, providing less invasive and quick diagnostic method. There are hardly any studies on the correlation between cytomorphology and prognostic biomarkers. We retrospectively analyzed the immunohistochemistry and the fluorescence in situ hybridization of breast cancer specimens from 252 patients, who have been diagnosed as breast cancer at our hospital. Morphological features of cytology smears were scored. The relationship between cytological features and three biomarkers were analyzed. Based on this, we developed a system to predict the status of biomarkers. The results indicated that some cytological parameters, especially the features of nucleoli, were distinctively related to the makers' expression. In the novel scoring system, a cutoff of 12.0 provided a statistical discrimination for cytological grading. We concluded that cytomorphological features were associated with prognostic factors. The HR+ neoplasms showed scattered micronucleoli, while HER2+ neoplasms demonstrated centered macronucleoli. We summarized a scoring system to predict the status of three factors. This may help us to broaden the application of breast cancer cytology.

## 1. Background

Breast cancer is one of the most common malignant diseases and remains the leading cause of cancer-related death in women [1]. Timely diagnosis is critical. Among the diagnostic and prognostic factors, estrogen receptor (ER), progesterone receptor (PR), and human epidermal growth factor receptor (HER2) play a significant role in guiding treatments. Hormone receptor (HR), including ER and PR, can regulate the growth and differentiation of normal breast epithelial cells, as well as tumor cells [2]. Patients with ER-positive and PR-positive neoplasms can benefit from hormonal therapies [3]. HER2 is a proto-oncogene, which can regulate cellular functions, such as proliferation and apoptosis. HER2-positive breast cancer is sensitive to targeted therapies, such as trastuzumab, lapatinib, and pertuzumab [4]. In clinical practice, it often takes 24 to 48 hours to get the results of immunohistochemistry and fluorescence in situ hybridization (FISH), after the biopsy or surgical resection.

Unlike histology, cytology focuses on analyzing cell structures to diagnose disease. Compared with needle core biopsy, ultrasound-guided fine needle aspiration cytology (FNAC) has gained wide-spread acceptance as a rapid, simple, and less invasive diagnostic method of breast mass [5]. However, the application of FNAC in breast cancer is limited to categorizing the tumor cells from tumor sample. The cytomorphological parameters from FNAC are merely used as a tool for cytological grading [6]. Its application needs to be expanded urgently. International consensus conferences on breast cancer have proposed to include prognostic factors in cytology reports [7].

It is well-known, histologically; the evaluation of the cytological features of breast cancer mainly depends on the Elston-Ellis modification of Scarff-Bloom-Richardson grading system, in which the tubule formation is one of the critical features. In contrast, cytologically, the tubule formation cannot be accurately assessed on cytological smears, while the cytological features can be better observed. In the absence of formaldehyde fixation and section, the morphology observation is closer to the true state of cells. The genetic expression changes of ER, PR, and HER2 largely depend on transcript levels and the expression of protein. It may also lead to cytomorphological changes [8]. Thus, discovering the distinctive cytological features of breast cancer cells may help pathologists enrich their understanding of the cases with different status of prognostic factors. To our best knowledge, few studies on the correlation between cytomorphological features of breast cancer and expression of prognostic factors are available. Previous studies mainly focused on application of cytological features for cytological grading, such as the widely-used Robinson's grading system and Taniguchi system [9, 10].

In this study, we found that some cytological features were related to the expression of ER, PR, and HER2, which launched the step stone for us to evaluate various parameters, aiming to find indicators related to prognostic factors. Moreover, the selected parameters were used to form an evaluation system to provide more information for cytological evaluation of breast cancer.

## 2. Materials and Methods

### 2.1. Case Selection

The study cohort included 252 female patients with invasive breast cancer, who were diagnosed at Sichuan Cancer Hospital & Institute between September 2018 and August 2019. The study was approved by ethics committee of Sichuan Cancer Hospital. The median age was 50-years old. All the patients were performed with ultrasound-guided FNAC of breast tumor before surgical treatment. The specimens of breast tumor were stained with immunohistochemistry for ER, PR, and FISH for HER2. Data of clinicopathological parameters, including age, tumor size, histological grade, histological subtype, and nodal involvement, were obtained from medical records.

### 2.2. Evaluation of Cytomorphological Features

Using a 20-gauge, FNAC was performed with the guidance of ultrasound. The cytology smears were fixed in 95% alcohol, then performed with routine hematoxylin and eosin (H&E) staining. According to the Robinson's and Taniguchi's grading system, two experienced pathologists, who were blinded to cytology diagnosis and histopathology diagnosis, evaluated eight standard cytological parameters independently. The cytology smears included dissociation, cell size, nuclear pleomorphism, nucleoli, nuclear margin, nuclear/cytoplasmic ratio, density of chromatin, and chromatin granularity [9, 11]. For each sample, the minimal cellularity criteria were 6 groups of at least 10 cells. We also analyzed three new parameters according to our observation: (a) nucleolar size: micro, medium, and macro; (b) nucleolar location: scattered and centered; and (c) nucleolar number: one, two-three, and more than three.

### 2.3. Immunohistochemical Analysis

HE and immunohistochemistry of breast mass from surgical resection were reviewed by two pathologists. The rabbit monoclonal antibodies included anti-ER (SP1, Maxim, Fuzhou, China), anti-PR (SP2, Maxim), and anti-C-erbB-2/HER2 (EP3, Maxim). All procedures were performed in the EnVision System by a Benchmark-ULTRA automatic immunohistochemical staining instrument (Asia-core, China). ER and PR were considered positive when there was more than 1% nuclear staining, according to the guideline of American Society of Clinical Oncology/College of America Pathologists (ASCO/CAP) [12, 13]. HER2 was considered negative (HER2−) with scores of 0 and 1 (no staining or <10% membrane staining of tumor cells) and positive (HER2+) with score of 3 (strong complete membrane staining in >10% tumor cells). Cases with score of 2 were considered positive only if FISH proved to be amplified.

### 2.4. Fluorescence in Situ Hybridization (FISH)

The test of HER2 was performed by using PanthVysion kit (GSP, LBP, Guangzhou, China). According to the guidelines of 2007 ASCO/CAP, the evaluation of HER2-amplification was based on the ratio of HER2 to centromere 17 copy number [14]. A case was considered as gene amplified for the HER2/CEP17 ratio of 2.2, negativity with the ratio less than 1.8 and equivocal with ratio less than 2.2 but more than 1.8. The equivocal cases were not selected in our cohort.

### 2.5. Statistical Analysis

SPSS (version 22) was used to analyze the data. The relationship between cytological parameters and the status of ER, PR, and HER2 was evaluated, using the Mann–Whitney *U* test, chi-square test, and Fisher's exact test. The scoring system was based on the regression model by valuating the predictive cytological parameters, according to the magnitude of each parameter estimate. *P* value <0.05 was considered statistically different.

## 3. Results

### 3.1. Clinicopathologic Characteristics of Invasive Breast Cancer

The clinical features of patients were shown in Table 1. Most cases (78.5%) were invasive carcinoma of no special type. Mean tumor size was 33 mm ranging from 6 to 150 mm. As for histologic grade, 65.9% was high. In addition, 143 cases (56.7%) were classified as T2. Results of ER, PR, and HER2 expression were summarized as follows: 58.7% were ER-positive, 53.2% were PR-positive, and 46% were HER2-overexpression, respectively. Specially, 85% ER positive cases had PR expression.

### 3.2. Cytomorphological Features with ER+, PR+, and HER2-Overexpression Tumors

**Figure 1:**
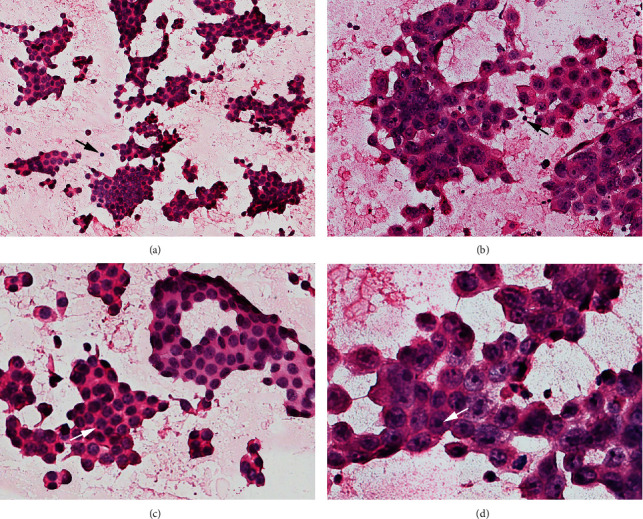
ER+ and/or PR+ tumor cells and HER2-overexpression tumor cells of breast cancer. (a) (×200) and (b) (×200) were the same magnification (black arrow showed lymphocytes were the same size in (a) and (b)). The nuclei of ER+ and/or PR+ tumors (a) were smaller than HER2-overexpression tumors (b), (c) (×400), and (d) (×400) were the same magnification. (c) In ER+ and/or PR+ tumor cells, the nucleoli accounted for about 1/5 of the nuclear diameter and was dispersed or clinging to the nuclear membrane (total score: 8). (d) In HER2-overexpression tumor cells, the nucleoli accounted for about 1/3 of the nuclear diameter and was located in the center of the nuclei (total score: 18). The scoring system was shown in Table 3.

The nuclei of ER+ and/or PR+ tumors (Figure 1(a)) were smaller than HER2-overexpression tumors (Figure 1(b)). In ER+ and/or PR+ tumor cells (Figure 1(c)), the nucleoli accounted for about 1/5 of the nuclear diameter and were dispersed or clinging to the nuclear membrane. In HER2-overexpression tumor cells (Figure 1(d)), the nucleoli accounted for about 1/3 of the nuclear diameter and were located in the center of the nuclei.

### 3.3. Correlation of Cytomorphological Features with Status of ER, PR, and HER2

To explore the relationship between cytological features and prognostic factors, we analyzed 11 parameters. The results were summarized in Table 2. Among the eight standard parameters, dissociation and nuclear margin were from Robinson's system, and nuclear/cytoplasmic ratio and density of chromatin were from Taniguchi's system. Besides, three new parameters including nucleolar size, nucleolar location, and nucleolar number were evaluated, based on our observation. In 252 samples, ER+ tumors had smaller cell than ER− tumors (*P* < 0.001). ER− tumors showed more marked pleomorphism (*P* < 0.001), nucleoli (*P* < 0.001), and folds, irregular nuclear margin (*P* < 0.001) than its competitor. In addition, significant difference was observed among PR+ and PR− samples.

There were two cytological parameters demonstrated statistically different between HER2-overexpression and HER2- samples. Larger cells and marked nuclear pleomorphism were detected in HER2+ samples (*P* < 0.001) (Figure 1(b)). No significant difference was observed between positive and negative samples of the markers when we tested the cytological features, which included dissociation, nuclear/cytoplasmic ratio, density of chromatin, and chromatin granularity.

Both ER+ and PR+ tumors showed smaller nucleolar size and less pleomorphism, compared to negative ones (*P* < 0.001). Additionally, in 106 ER+ and 98 PR+ tumors, the nucleolar location was scattered by contrast to 6 ER− samples and 14 PR− samples (*P* < 0.001). ER− or PR− tumors were more likely to show one or less nucleoli, compared with ER+ (*P* < 0.001) or PR+ (*P* < 0.001) tumors. In contrast, HER2+ samples had bigger nucleolar size (*P* < 0.001) but less cells than HER2− samples (*P* < 0.001). Besides, the nucleoli showed more centered in 92 (79.3%) HER2+ than HER2− samples (*P* < 0.001).

### 3.4. Multivariate Analysis and Cytological Model for Cytological Grading and Predicting Status of the Three Factors

Based on our findings, we finally selected six parameters, which were significantly associated with three biomarkers, to form a novel system. Detailed standards were shown in Table 3. For Figure 1(c), the total score of ER+ tumor cells were 8: The cell size scored 1. The nucleoli were indistinct, scoring 2. The nucleolar size was micro, scoring 1. The nucleolar location was scattered, scoring 1. The nucleolar number was 2–3, scoring 2. The nucleolar margin was smooth, scoring 1. For Figure 1(d), the total score of HER2-overexpression tumor cells were 18: The cell size scored 3. The nucleoli were prominent, scoring 3. The nucleolar size was macro, scoring 3. The nucleolar number was 1, scoring 3. The nucleolar location was centered, scoring 3. The nucleolar margin was irregular, scoring 3.

We next performed a multivariate analysis to identify the cytological features which were independently associated with the expression of ER, PR, and HER2 (Table 4). The results indicated that the cell size (*P* < 0.001), nucleolar size (*P* = 0.030), nucleolar location (*P* = 0.005), nucleolar number (*P* = 0.043), and nuclear margin (*P* = 0.043) were the independently associated with the expression of ER. In addition, only the cell size and nucleolar location were correlated with the status of PR and HER2.

The cutoff value was set as 12.0, according to the multivariate analysis. The score of 12.0 or lower was also regarded as a highly possibility with the expression of ER or PR upon the Receiver Operating Characteristic (ROC) analysis. At this cutoff (Table 5), the sensitivity and specificity were 94% and 77% for detecting the ER-expression. We also verified the scoring system for the expression of PR, the sensitivity was 86% and the specificity was 78%, respectively. With regard of HER2 status, the sensitivity, specificity, positive predictive value (PPV), and negative predictive value (NPV) were 76%, 68%, 67%, and 77%, respectively.

## 4. Discussion

In our cohort analysis, the cytomorphological features were strongly associated with the expression of ER, PR, and HER2. The cytological features of HR+ tumors were opposite with the HER2-amplication tumors. HR+ tumors showed scattered micronucleoli, while HER2-amplication tumors demonstrated centered macronucleoli and folds nuclear margins. The diametrically reserve cytological characteristics were probably due to the negative correlation between the expression of HR and HER2, which was conquered by previous studies [15, 16]. In detail, ER is composed of nuclear receptors (ER*α* and ER*β*) and membranous receptor. The existing evidence demonstrated that ER*β* partly counteracted the transcriptional and proliferative functions of ER*α* [17, 18]. ER*α* played a slow “genotype” regulatory to activate signaling pathways and promote cell proliferation [19], thus ER+ tumors showed slow proliferation of micronucleoli. Furthermore, estrogen binds to receptors which located on the nuclear membrane. Their combination could regulate gene expression. This may explain why we observed that the nucleolus is scattered but closed to the nuclear membrane. On the other hand, because of its impact on proliferation, HER2 was proved to be related to high nuclear levels and large tumor size, as well as high risk of recurrence and metastasis [20]. This high nuclear level was in accord with macronucleoli in HER2-amplification samples.

Based on our findings, we combined previous grading system with our new parameters, and selected six parameters, including cell size, nucleoli, nucleolar size, nucleolar location, nucleolar number, and nuclear margin. The new combined system was performed for further analysis. According to the logistic regression analysis, our system emphasis more on cytological features of nucleoli, such as nucleolar size (*P* = 0.03), nucleolar location (*P* = 0.004), and nucleolar number of ER (*P* = 0.043). Nuclei are essential for DNA replication or transcription, and nucleoli are a major part of it. The characteristics of nucleoli have been descripted in a research conducted by Kashyap et al. It demonstrated that nucleoli features were extremely important in differentiating cytological grades of malignant tumors [21]. Because the three biomarkers represented the proliferation of tumor cells, the changes in chromatin were not statistically different. This finding was consistent with prior study [22]. By investigating FNAC samples from patients with basal-like breast cancer, Akashi et al. found that among the cytology features of triple-negative (ER−, PR−, and HER2−) breast cancer, the nuclear size and margin were distinctive, while the features of chromatin were not statistically different among their cases.

Our scoring system was a novel one which combined cytological features with prognostic biomarkers. Most ER+ samples had a score of less than 12.0, while most HER-amplification samples had a score higher than 12.0. In our system, higher score was associated with higher grade of atypia. The atypical feature of HER2-overexpression tumors may be one of the reasons for its poor prognosis. Some studies have demonstrated that HER2 phenotype was a prognostic factor for poor outcomes [23]. Compared with HER2-overexpression tumors, ER-positive tumors have less effect on proliferation, and it is typically used to guide hormone therapy. Therefore, highly atypia cytomorphology with high score was more frequent appeared in HER2-overexpression tumors, rather than ER-positive tumors. In addition, by using a preclinical model of breast cancer, previous studies demonstrated that over-expression of HER2 leads to increasing cross-talk between ER and HER2 pathways, even if the mechanism of the influence between ER and HER2 remains unclear [24, 25].

At the same time, this system has a higher sensitivity for evaluating the status of ER (94%) and PR (86%), but much lower for HER2 (76%). Although the multivariate analysis shows a lower sensitivity in HER2-positive neoplasms, the specificity, sensitivity, positive predictive value (PPV), and negative predictive value (NPV) are better than Robinson's and Taniguchi's. In cytology, there have been at least six grading systems consist of cytomorphological features since 1980 [26–29]. In particular, Robinson's system is the most widely used, and Taniguchi's system is the only one which demonstrated the high grade was negatively correlated with ER status. On the other hand, Khan's study added the mitotic count to his system, which was not seen in most of our samples [29]. Therefore, regardless of the six systems' crucial role in cytological grading, our system seems to be the most optimal in predicting the status of ER, PR, and HER2.

It is true that our system has good sensitivity and specificity towards ER and PR, but it also has limitations of evaluating the status of HER2. This is because breast cancer with poor prognosis is related to several factors, which usually shows evident macronucleoli and high proliferation index in most times. For example, some genetic changes were demonstrated in breast cancer, such as the mutation of BRCA1/2 and TP53 [30]. Moreover, a histone variant consisting of C-terminal macro domain-MacroH2A1 was confined with breast cancer with worst prognosis and high risk of metastasis, which was same with HER2+ neoplasms [31]. We need further work to verify them.

Our system is suitable for epithelial breast cancer. However, there are various forms of breast neoplasms (epithelial, mesenchymal, and mixed). We need aware of mesenchymal and mixed breast neoplasms when dealing with a spindle cell lesion of the breast. The neoplastic cells of the breast carcinoma may adopt a spindled morphology raising confusion with benign/low-grade mesenchymal lesions [32]. For instance, fat necrosis is a diagnostic clue of reactive spindle cell nodule/exuberant scar; spindle cells are arranged in a swirling growth pattern in inflammatory pseudotumor; neoplastic cells may exhibit nuclear atypia in myxoma. We should be vigilant against the existence of these neoplasms and make further diagnosis by biopsy and immunohistochemistry. For example, neoplastic cells are diffusely stained with CD34 in dermatofibrosarcoma protuberans.

## 5. Conclusions

In summary, we found that scattered micronucleoli was in HR+ tumor, while centered macronucleoli and folds nuclear margins appeared in HER2-amplication tumor. Based on this, we summarized six parameters to form a scoring system. Our analysis demonstrated that the system is a novel one to predict the status of three factors. This may help us to broaden the application of breast cancer cytology.

## Figures and Tables

**Table 1:** Clinicopathologic features of all patients suffering from breast carcinoma (*N* = 252).

Clinicopathologic parameters	Number of sample (%)
*Age (years) (mean 50, median 49, range 25–76)*	
<40	23 (9.1%)
40 to 50	124 (49.2%)
>50	105 (41.7%)
*Tumor size (mm) (mean 33, median 30, range 6–150)*	
<20	41 (16.3%)
20 to 50	174 (69.0%)
>50	19 (7.5%)
Unknown	18 (7.1%)
*Histologic grade*	
1	6 (2.4%)
2	73 (29.0%)
3	166 (65.9%)
Not assessable	7 (2.8%)
*Expression of ER, PR and HER2 (positive/negative)*	
ER (+/−)	148/104(58.7%/41.3%)
PR (+/−)	134/118(53.2%/46.8%)
HER2(+/−)	116/136(46.0%/54.0%)
*Histologic subtype*	
IDC (NOS)	198 (78.5%)
ILC	24 (9.6%)
Other invasive breast carcinomas	25 (9.9%)
Unknown	5 (2.0%)
*Lymph node status*	
Metastasis in 1 to 3 lymph nodes	159 (70.6%)
Metastasis in 4 or more lymph nodes	60 (23.8%)
Not available	14 (5.6%)
*Stage*	
T1	35 (13.9%)
T2	143 (56.7%)
T3	41 (16.3%)
T4	20 (8.4%)
Unknown	13 (5.2%)

**Table 2:** Correlation between cytological parameters (eight standard and three new parameters) and the status of ER, PR, and HER2.

	ER			PR			HER2		
	Positive	Negative	*P* value	Positive	Negative	*P* value	Positive	Negative	*P* value
*Dissociation*			0.971			0.508			0.037
Clusters	34(23.0)	28(26.9)		33(24.6)	29(24.6)		26(22.4)	36(26.5)	
Single and clusters	76(51.4)	45(43.3)		68(50.7)	53(44.9)		49(42.2)	72(52.9)	
Single	38(25.7)	31(29.8)		33(24.6)	36(30.5)		41(35.3)	28(20.6)	
*Cell size*			<0.001			<0.001			<0.001
1–2 × RBC	52(35.1)	7(6.7)		48(35.8)	11(9.3)		12(10.3)	47(34.6)	
2–4 × RBC	84(56.8)	46(44.2)		71(53.0)	59(50.0)		57(49.1)	73(53.7)	
≥5 × RBC	12(8.1)	51(49)		15(11.2)	48(40.7)		47(40.5)	16(11.8)	
*Nuclear pleomorphism*			<0.001			<0.001			<0.001
Uniform	25(16.9)	5(4.8)		23(17.2)	7(5.9)		6(5.2)	24(17.6)	
Mild	118(79.7)	71(68.3)		104(77.6)	85(72.0)		87(75.0)	102(75.0)	
Marked	118(79.8)	28(26.9)		7(5.2)	26(22.0)		23(19.8)	10(7.4)	
*Nucleoli*			<0.001			<0.001			0.09
Indistinct	53(35.8)	10(9.6)		46(34.3)	17(14.4)		24(20.7)	39(28.7)	
Noticeable	69(46.6)	60(57.7)		65(48.5)	64(54.2)		60(51.7)	69(50.7)	
Prominent	26(17.6)	34(32.7)		23(17.2)	37(31.4)		32(27.6)	28(20.6)	
*Nuclear margin*			<0.001			0.004			0.391
Smooth	9(6.1)	5(4.8)		9(6.7)	5(4.2)		7(6.0)	7(5.1)	
Folds	133(89.9)	71(68.3)		115(85.8)	89(75.4)		90(77.6)	114(83.8)	
Buds	6(4.1)	28(26.9)		10(7.5)	24(20.3)		19(16.4)	15(11.0)	
*Nuclear/cytoplasmic ratio*			0.507			0.844			0.517
<50	8(5.4)	15(14.4)		8(6.0)	15(12.7)		13(8.9)	10(9.4)	
50–80	126(85.1)	74(71.2)		114(85.1)	86(72.9)		115(78.8)	85(80.2)	
>80	14(9.5)	15(14.4)		12(9.0)	17(14.4)		18(12.3)	11(10.4)	
*Density of chromatin*			0.13			0.001			0.778
Not	44(29.7)	43(41.3)		33(24.6)	54(45.8)		43(37.1)	44(32.4)	
Moderately	97(65.5)	54(51.9)		93(69.4)	58(49.2)		64(55.2)	87(64.0)	
Markedly	7(4.7)	7(6.7)		8(6.0)	6(5.1)		9(7.8)	5(3.7)	
*Chromatin granularity*			0.853			0.214			0.443
Fine	80(54.1)	58(55.8)		76(56.7)	62(52.5)		62(53.4)	76(55.9)	
Moderately granular	35(23.6)	17(16.3)		32(23.9)	20(16.9)		21(18.1)	31(22.8)	
Coarse	33(22.3)	29(27.9)		26(19.4)	36(30.5)		33(28.4)	29(21.3)	
*Nucleolar size^#^*									
Micro	115(77.7)	17(16.3)	<0.001^a^	106(79.9)	26(22.0)	<0.001	39(33.6)	94(69.1)	<0.001
Medium	31(21.0)	63(60.6)	<0.001^b^	21(15.7)	74(62.7)		62(53.4)	33(24.3)	
Macro	2(0.01)	24(23.1)		7(4.5)	18(15.3)		15(12.9)	9(6.6)	
*Nucleolar location^#^*			<0.001^c^			<0.001^c^			<0.001^c^
Peripheral	106(71.6)	6(5.8)		98(73.1)	14(11.9)		24(20.7)	88(64.7)	
Centered	42(28.4)	98(94.2)		36(26.9)	104(88.1)		92(79.3)	48(35.3)	
*Nucleolar number^#^*			<0.001			<0.001			<0.001
≤1	61(41.2)	97(93.3)		54(40.3)	104(88.1)		94(81.0)	64(47.1)	
2–3	32(21.6)	5(4.8)		31(23.1)	6(5.1)		9(7.8)	28(20.6)	
>3	55(37.2)	2(1.9)		49(36.6)	8(6.8)		13(11.2)	44(32.4)	

Fisher's exact test (mico vs medium and macro). Fisher's exact test (medium vs macro). chi-square test (peripheral vs centered) and others were tested with Mann–Whitney *U* test. #: new parameters from our observation.

**Table 3:** The scoring system for predicting the status of ER, PR, and HER2.

	Score 1	Score 2	Score 3
Cell size	<3 × RBC size	3–4 × RBC size	>4 × RBC size
Nucleoli	Indistinct	Noticeable	Prominent
Nucleolar size	Micro	Medium	Macro
Nucleolar location	Scattered		Centered
Nucleolar number	>3	2–3	≤1
Nucleolar margin	Round and smooth	Smooth	Irregular

**Table 4:** Multivariate logistic regression analysis for determination of the likelihood of ER, PR, and HER2 status from system's cytological features.

	ER+		PR+		HER2+	
	OR (95% CI)	*P* value	OR (95% CI)	*P* value	OR (95% CI)	*P* value
Cell size	0.237 (0.122–0.460)	<0.001	0.469 (0.277–0.791)	0.005	2.548 (1.583–4.104)	<0.001
Nucleoli	0.565 (0.303–1.052)	0.072	0.688 (0.414–1.145)	0.15	0.992 (0.638–1.543)	0.992
Nucleolar size	0.425 (0.196–0.921)	0.03	0.678 (0.337–1.3255)	0.271	0.861 (0.457–1.624)	0.65
Nucleolar location	6.201 (1.758–21.878)	0.005	6.889 (2.546–18.638)	<0.001	0.224 (0.090–0.563)	0.001
Nucleolar number	0.363 (0.136–0.969)	0.043	0.697 (0.377–1.289)	0.25	1.253 (0.746–2.104)	0.394
Nuclear margin	0.367 (0.139–0.967)	0.043	0.890 (0.402–1.969)	0.773	0.761(0.378–1.533)	0.445

OR: odds radio.

**Table 5:** Sensitivity, specificity, PPV, and NPV from the three system for assessment the status of ER, PR, and HER2.

	Sensitivity			Specificity			PPV			NPV		
	ER	PR	HER2	ER	PR	HER2	ER	PR	HER2	ER	PR	HER2
Our system	0.94	0.86	0.76	0.77	0.78	0.68	0.74	0.77	0.67	0.95	0.87	0.77
Robinson system	0.55	0.57	0.72	0.76	0.75	0.55	0.77	0.72	0.58	0.54	0.61	0.7
Taniguchi system	0.61	0.58	0.63	0.62	0.56	0.64	0.69	0.6	0.6	0.52	0.54	0.67

PPV: positive predictive value; and NPV: negative predictive value.

## Data Availability

The data used to support the findings of this study are available from the corresponding author upon request.
